# The complete chloroplast genome of *Pimpinella rhomboidea* var. *tenuiloba*

**DOI:** 10.1080/23802359.2018.1424580

**Published:** 2018-01-09

**Authors:** Jinbo Tan, Yan Yu

**Affiliations:** Key Laboratory of Bio-Resources and Eco-Environment of Ministry of Education, College of Life Sciences, Sichuan University, Chengdu, P. R. China

**Keywords:** Chloroplast, genome, angelica, *Pimpinella*

## Abstract

*Pimpinella rhomboidea* var. *tenuiloba* is an endemic species in China. The complete chloroplast genome sequence of *P. rhomboidea* var. *tenuiloba* was generated by *de novo* assembly using whole genome next generation sequencing. The genome was 146,655 bp in length, composed of four distinct regions such as large single copy (LSC) region of 94,684 bp, small single copy (SSC) region 17,537 bp and a pair of inverted repeat regions of 17,217 bp. The genome annotation predicted a total of 113 genes, including 80 protein-coding genes, 29 tRNA genes, and 4 rRNA genes. Phylogenetic analysis with the reported chloroplast genomes revealed that *P. rhomboidea* var. *tenuiloba* has close relationship with genus *Angelica*.

*Pimpinella rhomboidea* Diels var. *tenuiloba* Shan & Pu (Apiaceae, Apioideae), a perennial herb which is endemic to mountainous region of West Sichuan, China, growing at an elevation of 2200–4000 m (Tan et al. 2015). Based on our previous work (Tan et al. 2015), *P. rhomboidea* var. *tenuiloba* was treated as a synonym of *Melanosciadium bipinnatum* (Shan and Pu) Pimenov & Kljuykov, and has been corroborated to nest in *Angelica* sensu lato within Selineae (Zhou et al. 2009, Liao et al. 2013, Wang et al. 2014). Here, we assembled the complete chloroplast genome of *P. rhomboidea* var. *tenuiloba*, to explore the reasonably systematic position of this taxon.

The mature leaves of *P. rhomboidea* var. *tenuiloba* were collected from Mt. Balang (30°53′55.19″N 102°54′38.06″E), Sichuan Province, China. Voucher specimens were deposited in SZ (Sichuan University Herbarium). The isolated genomic DNA was manufactured to average 350bp paired-end (PE) library using the Illumina Hiseq platform, and sequenced by Illumina genome analyzer (Hiseq PE150). Chloroplast genome-related reads were sieved by mapping to all existent chloroplast genomes of Apioideae in GenBank using Bowtie2. Contigs, assembled using SOAPdenovo2 (Luo et al. 2012) and SPAdes (Bankevich et al. 2012), were sorted and joined into a single-draft sequence using Geneious 11.04 (Kearse et al. 2012) by comparison with the chloroplast sequences of *Angelica*. The draft sequence was then corrected manually by clean reads mapping using bowtie2 (Langmead and Salzberg 2012) and Tablet (Milne et al. 2013). The genes in chloroplast genome were predicted using Geneious by pairwise align with sequence of *Angelica gigas*, and revised manually.

The chloroplast genome of *P. rhomboidea* var. *tenuiloba* (Genbank accession no. MG719855) was a circular molecular genome with a size of 146,655 bp in length, which was composed of four distinct regions such as large single copy (LSC) region of 94,684 bp, small single copy (SSC) region 17,537 bp and a pair of inverted repeat regions of 17,217 bp. The overall GC content was 37.5%. The chloroplast genome contained 80 protein-coding genes, 29 tRNA genes and 4 rRNA genes.

In order to understand the phylogenetic relationship between *P. rhomboidea* var. *tenuiloba* and related species, the complete chloroplast genome sequences of 12 genera (16 species) from apioid superclade and one outgroup (*Daucus carota*) were aligned by MAFFT (Katoh et al. 2002) and trimmed properly by trimAl v1.4 (Capella-Gutierrez et al. 2009). The evolutionary history was inferred by using the Neighbour-joining method in MEGA7.0 (Kumar et al. 2016). The percentage of replicate trees in which the associated taxa clustered together in the bootstrap test (100,000 replicates) are shown next to the branches (Figure 1). As was expected, *P. rhomboidea* var. *tenuiloba* was placed within *Angelica* species, and occurred as a sister to the clade of *A. dahurica*, *A. nitida* and *A. gigas* with 100% BS value.

**Figure 1.**
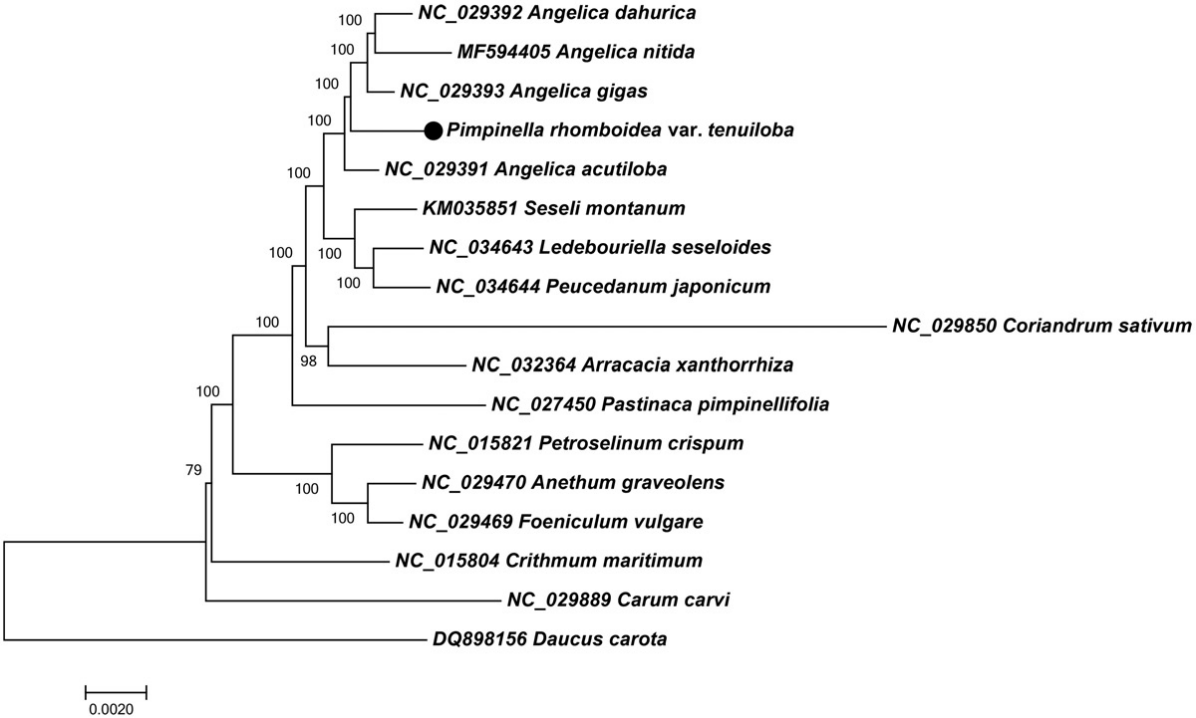
Neighbour-joining tree of *P. rhomboidea* var. *tenuiloba* and related species using chloroplast sequences. Numbers on the nodes are bootstrap values from 100,000 replicates. *Daucus carota* was used as outgroup.
